# Supplementary material for: Inhibition of Candida albicans and Mixed Salivary Bacterial Biofilms on Antimicrobial Loaded Phosphated Poly(methyl methacrylate)
Source: Antibiotics (Basel). 2021 Apr 13;10(4):427. doi: 10.3390/antibiotics10040427 (PMC8070037; doi:10.3390/antibiotics10040427)
Supplement: Supplementary file 1 [file antibiotics-10-00427-s001.pdf]

antibiotics

MDPI

Article

Inhibition of *Candida albicans* and Mixed Salivary Bacterial Biofilms on Antimicrobial Loaded Phosphated Poly(methyl methacrylate)

Andrew R. Dentino [1,*], DongHwa Lee [1], Kelley Dentino [1], Arndt Guentsch [1] and Mohammadreza Tahriri [2]

1 Department of Surgical Sciences, Marquette University School of Dentistry, Milwaukee, WI 53201-1881, USA; evergreenx@yahoo.com (D.L.); kelley.dentino@marquette.edu (K.D.); arndt.guentsch@marquette.edu (A.G.)
2 Department of Engineering, Norfolk State University, Norfolk, VA 23504, USA; mtahriri@nsu.edu
* Correspondence: andrew.dentino@marquette.edu

Figure S1: Charge-dependent inhibition of *C. albicans* strain 1.1 suggesting a plateau effect plateau between 15—25% Phosphated PMMA.

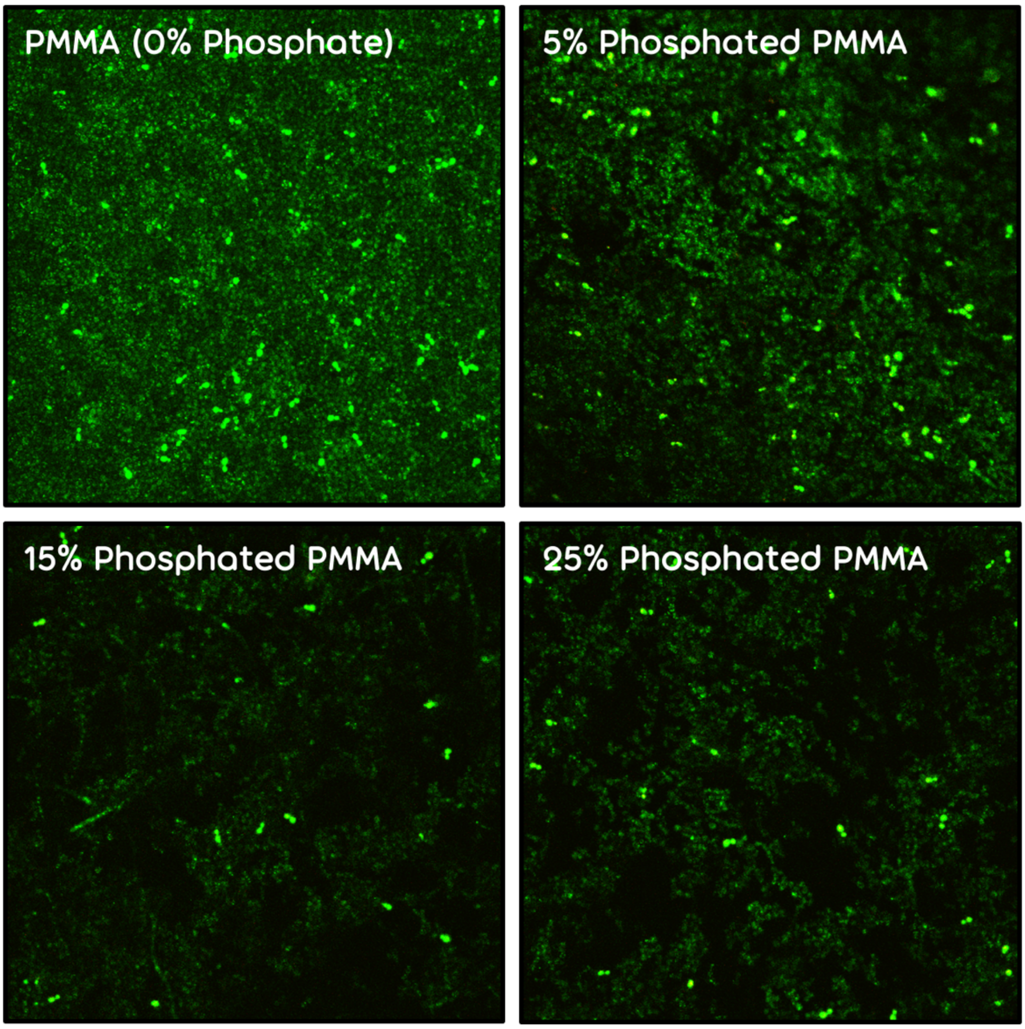

Figure S1. S. *C. albicans* in a 24 h biolfim model on PMMA (0%-25% Phosphate) under CSLM showing anti-adhesion and biofilm development due to charge alone. Alterations above 15% are minimal.

Figure S2: Effects of artificial saliva and/ or CPC on 4 strains of *C. albicans* on PMMA showing all 4 strains of *C. albicans* in a 24 h biofilm model on PMMA (0% Phosphate) under CSLM. Data suggest the 4 strains show minimal sensitivity to Biotene PBF pellicle alone, variable sensitivity to CPC pellicle alone, but all four strains are substantially inhibited when a protein pellicle (Biotene PBF) is present with CPC (350 µg/ml) prior to *C. albicans* inoculation.

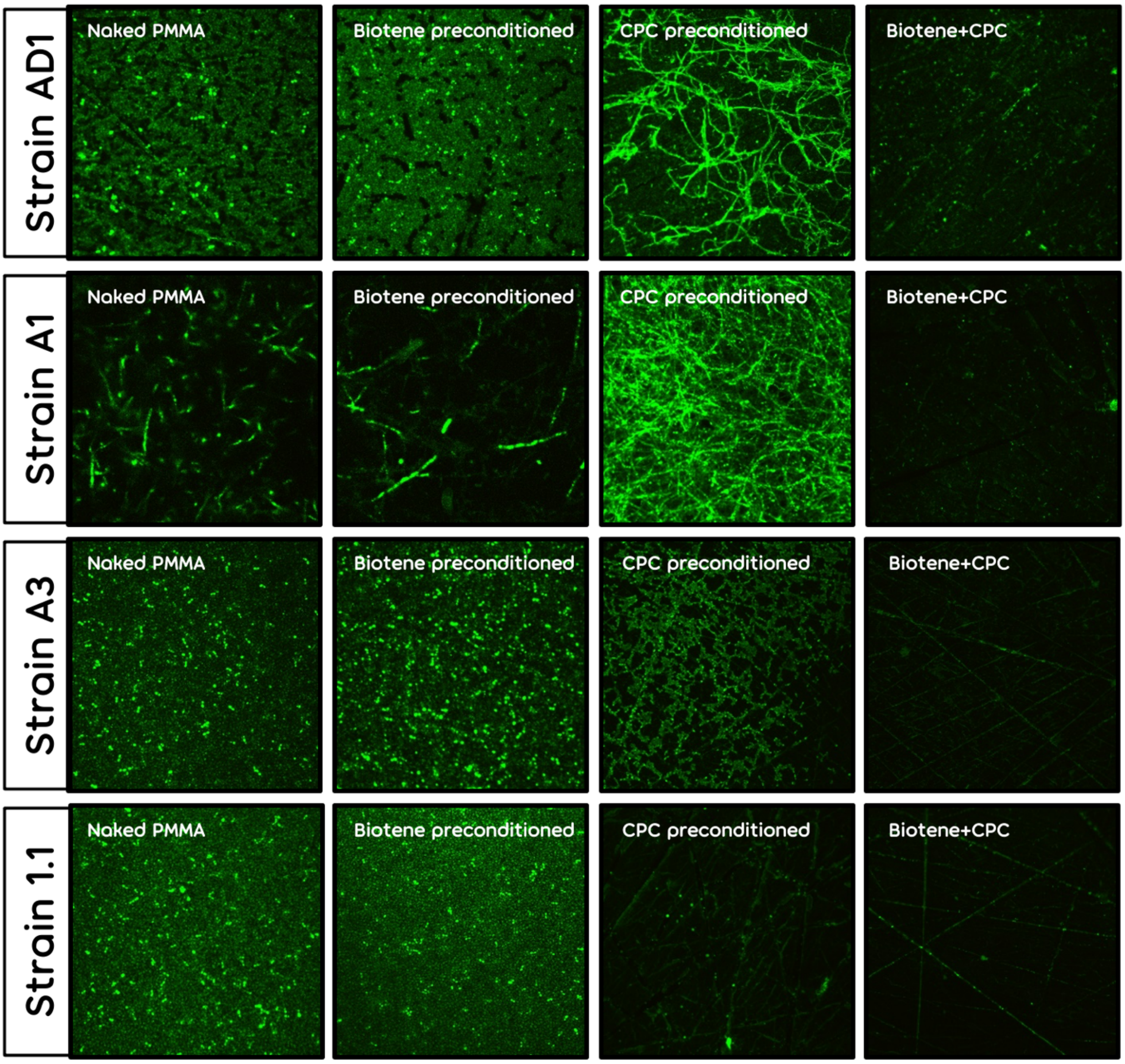

Figure S2. 4 strains of *C. albicans* in a 24 h biolfim model on PMMA (0% Phosphate) under CSLM. Strain variations can be seen in the Biotene only and the CPC only pellicles.
